# Evidence on risk factors for knee osteoarthritis in middle-older aged: a systematic review and meta analysis

**DOI:** 10.1186/s13018-023-04089-6

**Published:** 2023-08-29

**Authors:** Yawei Dong, Yan Yan, Jun Zhou, Qiujun Zhou, Hongyu Wei

**Affiliations:** 1 Beijing University of Chinese Medicine, Beijing, China; 2 Department of First Clinical Medical College, Zhejiang Chinese Medical University, No. 2, Sakura Garden East Street, Chaoyang District, Beijing, China; 3 Department of Orthopaedic Surgery, China-Japan Friendship Hospital, Beijing, China

**Keywords:** Knee osteoarthritis, Middle-older aged, Risk factors, Meta-analysis

## Abstract

**Purpose:**

This review was made to identify the risk factors for knee osteoarthritis (KOA) in middle-older aged (≥ 40 years), and to provide the newest evidence for the prevention of KOA.

**Method:**

Cohort study and case–control study of the risk factors of KOA was included from Pubmed, Web of Science, Ovid Technologies, China National Knowledge Infrastructure (CNKI), Chinese Science and Technology Periodical Database (VIP), Wanfang Database, SinoMed from their inceptions to July 2023. Two authors independently screened the literature and extracted data. Assessment of quality was implemented according to Agency for Healthcare Research and Quality (AHRQ) and Newcastle–Ottawa Quality Assessment Scale. Meta-analysis was performed using RevMan 5.3 software.

**Results:**

3597 papers were identified from the seven databases and 29 papers containing 60,354 participants were included in this review. Meta-analysis was performed for 14 risk factors, and 7 of these were statistical significance (*P* < 0.05). The risk factors which were analyzed in this review included trauma history in knee (1.37 [95% CI 1.03–1.82], *P* = 0.030), body mass index (BMI) ≥ 24 kg/m^2^ (1.30 [95% CI 1.09–1.56], *P* = 0.004), gender (female) (1.04 [95% CI 1.00–1.09], *P* = 0.030), age ≥ 40 (1.02 [95% CI 1.01–1.03], *P* = 0.007), more exercise (0.75 [95% CI 0.62–0.91], *P* = 0.003), a high school education background (0.49 [95% CI 0.30–0.79], *P* = 0.003) and an university education background (0.22 [95% CI 0.06–0.86], *P* = 0.030).

**Conclusion:**

The risk factors analyzed in this review included trauma history in knee, overweight or obesity, gender (female), age ≥ 40 and the protective factors included more exercise and a high school or an university education background.

**Supplementary Information:**

The online version contains supplementary material available at 10.1186/s13018-023-04089-6.

## Introduction

Knee osteoarthritis (KOA) is a prevalent disease, affecting an estimated 32.5 million adults in the US, with 14% of the American population experiencing symptomatic KOA between 2008 and 2014 [1]. The incidence of KOA is progressively increasing. Despite the high number of people affected by KOA, current treatment options primarily focus on symptom relief [2], with joint replacement being the ultimate outcome. This approach carries a significant financial burden and results in poor quality of life for patients. Early identification and avoidance of risk factors can alleviate the social burden associated with KOA by reducing its incidence. Therefore, it is imperative to identify the risk factors associated with KOA.

A systematic review and meta-analysis published in 2015 which aimed for the current evidence on risk factors for onset of knee pain/OA in those aged 50 and over identified a set of factors [3]. The factors were overweight, obesity, female gender and previous knee injury. Hand osteoarthritis (OA) was found to be non-significant. Which is different from other systematic review published in 2010 [4]. The reasons for the difference may be the limited evidence with case–control studies excluded, and the diverse inclusion and exclusion criteria. As the evidence of risk factors of knee osteoarthritis is updating, the newer evidence can give guidance for the risk of KOA.

The objective of this systematic review and meta-analysis was to determine the current evidence on risk factors for onset of KOA in middle-older aged. Patients can avoid these risk factors at home, thereby reducing the incidence of knee osteoarthritis (KOA) and providing a foundation for the development of KOA guidelines.

## Methods

This study has been registered in the PROSPERO (CDR42022329710), in accordance with the Preferred Reporting Items for Systematic Reviews and Meta-Analyses (PRISMA) criteria [5].

### Search strategy and study selection

The present study includes cohort and case–control studies on the risk factors of knee osteoarthritis (KOA) published from the commencement of database construction until July 2023. The databases searched include Pubmed, Web of Science, Ovid Technologies, China National Knowledge Infrastructure (CNKI), Chinese Science and Technology Periodical Database (VIP), Wanfang Database, and SinoMed. The search strategy utilized Medical Subject Headings (MeSH) terms and relevant keywords to identify studies that investigated the association between KOA and potential risk factors. Please refer to Additional file 1: Appendix 1 for the complete search strategy used in every databases.

Two authors(Y Yan and J Zhou) reviewed all abstracts independently, and a third author (YW Dong) will review those if a consensus had not been reached. Two authors then assessed all remaining papers for inclusion in the final review. Disagreements were resolved by consensus.

### Inclusion and exclusion criteria

#### Inclusion criteria

1. Chinese or English language.
2. Diagnostic Criteria for Knee Osteoarthritis in the included Studies: *American College of Rheumatology (ACR) guidelines* [6] or *Chinese guideline for diagnosis and treatment of osteoarthritis* [7] in Case–control study. The severity of knee osteoarthritis (KOA) was assessed using the *Kellgren–Lawrence (K–L)* grading system [8] in the cohort study.
3. Observational or cohort studies, and cases and controls were defined based on the presence of KOA (with or without).
4. Outcome of onset of knee OA, knee pain, knee disability or physical limitations relating to knee or radiographic knee OA.
5. Mean age at follow-up of 40 + or age stratified analysis with 40 + strata. (We found that the onset of the KOA occurs 10 years earlier in women than in men clinically, thus we studied middle-older aged more than 40 to obtain more complete risk factors.)
6. Risk factors that may contribute to the development of knee osteoarthritis e.g., demographic, socio-economic, comorbidity, previous knee events (for example injury) and other patient determined factors.
7. Sufficient data were published for estimating an odds ratio (OR) or weighted mean difference (WMD) with 95% confidence intervals (CIs).

#### Exclusion criteria

1. Knee pain related to other musculoskeletal conditions e.g., rheumatoid arthritis, rheumatism
2. Studies whose outcome is a total knee replacement or studies of patients following total knee replacements.
3. A study was also excluded if it was clearly not a comparative study.
4. Animal studies, Conference abstracts, not an original study (e.g., editorial, literature review, comments, letters, editorials, protocols, guidelines and review papers.)
5. Clinical risk factors or outcome including proprioception, muscle strength, joint alignment, cartilage loss.

### Data extractiong

The study included the following variables: first author, publication year, location, study type, journal, number of participants, age, sex, and definition of osteoarthritis (OA). Additionally, the paper's mentioned risk factors, such as gender, age, BMI, trauma history, family history, living environment, education background, occupation, alcohol consumption, smoking, comorbidity, and exercise, were extracted as outcomes.

Odds ratios (OR) for the association of each potential risk factor with knee osteoarthritis (KOA) were extracted from each paper. KOA was typically diagnosed clinically based on patient-reported symptoms of pain, stiffness, or reduced function. Radiographic-based OA was determined by an increasing Kellgren Lawrence (K/L) score or a K/L score of 2 or more [7]. Log (OR) and standard error (SE) were calculated based on the OR extracted from each paper.

### Assessment of quality

For cross-sectional study, the methodological quality of the studies included was assessed using an 11-item checklist which was recommended by Agency for Healthcare Research and Quality (AHRQ) [9, 10]. An item would be scored ‘0’ if it was answered ‘NO’ or ‘UNCLEAR’; if it was answered ‘YES’, then the item scored ‘1’. Article quality was assessed as follows: low quality = 0–3; moderate quality = 4–7; high quality = 8–11.

For case–control study and cohort study, Newcastle–Ottawa Quality Assessment Scale [9, 10] was used for assessing the quality of included study. There are three dimensions which are Selection, Comparability and Exposure, and when a particular item is met, a star will be gained. A study can be awarded a maximum of one star for each numbered item within the Selection and Exposure categories. A maximum of two stars can be given for Comparability.

In order to assess the quality of the meta-analysis results as a whole, a Grading of Recommendations Assessments, Development and Evaluation (GRADE) assessment was conducted meanwhile.

### Statistical analysis

Meta-analysis was conducted with RevMan version 5.3 (The Cochrane Collaboration, London, UK). We extracted adjusted ORs (from multi-variate analysis models) and its 95% CIs (Confidence Interval)) from the original studies. If studies presented both unadjusted ORs and ORs adjusted for potential confounders, we used adjusted ORs to assess the associations between different variables and the risk of KOA. The *I*^2^ statistic was calculated to assess the proportion of total variance accounted for by heterogeneity between studies, and values exceeding 50% implied moderate to high heterogeneity [11]. Given the non-uniformity of the research type in this article, we have opted for a random-effects model for analysis, even if the value of *I*^2^ is less than 50%.

For any variable presenting with large heterogeneity, a sensitive analysis excluding outlier studies was conducted to investigate the potential sources of heterogeneity. We attempt to assess the possibility of publication bias by constructing a funnel plot of each trial’s effect size against the standard error.

For meeting the purpose of this review, potential risk factors were searched by browsing the literatures [12–16]. To facilitate result analysis, subgroup analyses were conducted separately for cohort and case–control studies on certain risk factors. Overweight was defined as having a BMI of 24–28, while obesity was defined as having a BMI over 28. Living environment was classified as damp, dark, cold, or both damp and dark. Education background was categorized as junior high school, high school, or university. Manual labor and agricultural work were included as types of Working.

## Results

### Literature search and screening

**Fig. 1:**
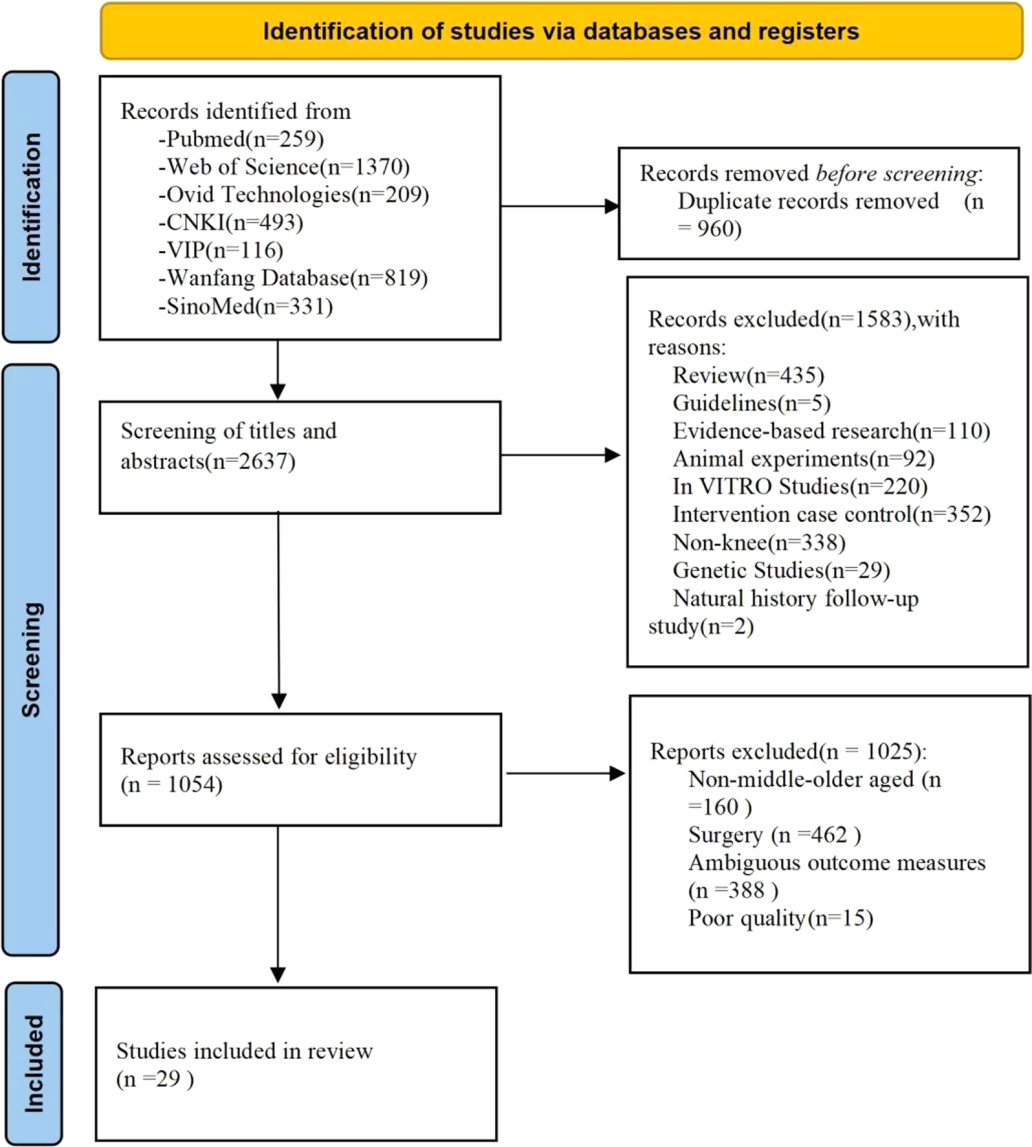
Flowchart of study selection

From the electronic search conducted, 3597 papers were identified from the seven databases, in total, 29 papers [12–40] (60,354 participants) were included in this review (Fig. 1).

### Assessment of quality

17 case–control studies and 12 Cohort studies were included in all of the 29 papers included in this review. A lack of Selection of Controls and incomplete information about the Non-Response rate were the most common shortcomings in these studies. The mean score across all included case–control studies was 5.7 and all included Cohort studies 7. Additional file 2: Appendix 2 and Additional file 3: Appendix 3 show detailed assessment using Newcastle—Ottawa Quality Assessment Scale and detailed GRADE assessment.

### Characteristics of the eligible studies

The 29 studies included in this review were performed in 11 different locations, and were published between 2005 and 2023. 17 case–control studies and 12 Cohort studies were included in this review which contains 60,354 participants. Risk factors contain trauma history, BMI ≥ 24 kg/m^2^, gender, Age, exercise, education background in high school, education background in university, education background in junior high school, Living environment, Agricultural labour, Manual labour, Family history, Drinking, and Smoking. The main characteristics of the trials are summarised in Table 1.

**Table 1:** Characteristics of the included studies

Study	Location	Study design (period)	Sample size (cases)	Female /male	Diagnostic guideline in KOA	Definition of OA	Risk factors
C Zhao [15]	China	Case–control	315	186/129	Development of criteria for the classification and reporting of osteoarthritis (ACR)	Symptomatic radiographic knee OA	Gender, Age, BMI, Family history, Smoking, Drinking
YS Lin [28]	China	Case–control	200	/	Development of criteria for the classification and reporting of osteoarthritis (ACR)	Symptomatic radiographic knee OA	BMI
HY Xu [32]	China	Case–control	578	352/221	Development of criteria for the classification and reporting of osteoarthritis (ACR)	Symptomatic knee OA	Family history, BMI
CH He [35]	China	Case–control	247	168/79	Development of criteria for the classification and reporting of osteoarthritis (ACR)	Symptomatic radiographic knee OA	Gender, Living environment
XS X [31]	China	Case–control	2880	1830/1050	Development of criteria for the classification and reporting of osteoarthritis (ACR)	Symptomatic radiographic knee OA	Gender, Age, BMI, Education background, Living environment, Family history, Trauma history, Exercise
JZ Zhao [24]	China	Case–control	250	/	Development of criteria for the classification and reporting of osteoarthritis (ACR)	/	Age, BMI, Living environment, Exercise, Working, Family history
YC Yang [22]	China	Case–control	285	240/45	Development of criteria for the classification and reporting of osteoarthritis (ACR)	Symptomatic radiographic knee OA	BMI, Smoking, Drinking, Trauma history, Exercise
GL Sun [16]	China	Case–control	263	149/114	Development of criteria for the classification and reporting of osteoarthritis (ACR)	Symptomatic radiographic knee OA	Age, Gender, Education background, Living environment, Exercise
SY Wang [18]	China	Case–control	147	72/75	Chinese guideline for diagnosis and treatment of osteoarthritis	Symptomatic knee OA	Gender, Age
FS Zhang [23]	China	Case–control	876	504/372	Chinese guideline for diagnosis and treatment of osteoarthritis	Symptomatic radiographic knee OA	Age, BMI, Gender, Working
Y Liu [17]	China	Case–control	415	287/128	Development of criteria for the classification and reporting of osteoarthritis (ACR)	Symptomatic radiographic knee OA	Age, Gender, Working, BMI
Moghimi [19]	Iran	Case–control	1400	/	Development of criteria for the classification and reporting of osteoarthritis (ACR)	Symptomatic knee OA	Age, Smoking, BMI, Comorbidity
Mounach [37]	Morocco	Case–control	190	52/138	Development of criteria for the classification and reporting of osteoarthritis (ACR)	Symptomatic radiographic knee OA	BMI
El Ayoubi [27]	Lebanon	Case–control	177	99/78	Development of criteria for the classification and reporting of osteoarthritis (ACR)	Symptomatic knee OA	Working, Family history
Jinks [38]	UK	Cohort	2059	1558/501	K–L	Symptomatic knee OA	BMI, Trauma history, Gender, Comorbidity
Takiguchi [20]	Japan	Cohort	11,058	5457/5601	K–L	Symptomatic radiographic knee OA	Age, Education background, Smoking, Working
Holmberg [39]	Sweden	Case–control	1473	/	Development of criteria for the classification and reporting of osteoarthritis (ACR)	Symptomatic radiographic knee OA	BMI
Klussmann [34]	Germany	Case–control	1310	569/741	Development of criteria for the classification and reporting of osteoarthritis (ACR)	Symptomatic knee OA	Smoking
Ingham [33]	UK	Cohort	2156	1181/975	K–L	Radiographic knee OA	Age, Gender, BMI, Trauma history
Muraki [29]	Japan	Cohort	2262	1499/763	K–L	Radiographic knee OA	Age, BMI, Gender, Smoking, Drinking
Huétink [25]	Netherlands	Cohort	319	106/213	K–L	Radiographic knee OA	Age, Gender, BMI, Family history,
Sanghi [26]	India	Case–control	360	/	Development of criteria for the classification and reporting of osteoarthritis (ACR)	Radiographic knee OA	Age, BMI, Gender
Sasaki [13]	Japan	Cohort	1014	/	K–L	Radiographic knee OA	Gender, Working, Exercise, Smoking, Drinking, age, BMI
Ito [14]	Japan	Cohort	7434	/	K–L	Symptomatic knee OA	Age, BMI, Somking, Drinking
Yoshimura [30]	Japan	Cohort	1384	918/466	K–L	Radiographic knee OA	BMI, Comorbidity
Konstari [12]	Finland	Cohort	6274	/	K–L	Symptomatic knee OA	Gender, BMI, Smoking, Working, Trauma history, Comorbidity,
Wang [40]	China	Cohort	8193	4251/3942	K–L	Symptomatic knee OA	Gender, Living environment, Smoking, Comorbidity
Konstari [21]	Finland	Cohort	4553	2290/2263	K–L	Symptomatic knee OA	Gender, Age, Exercise, Trauma history,
Muraki [36]	Japan	Cohort	2282	1465/817	K–L	Radiographic knee OA	Age, Gender, BMI, Living environment,

### Meta-analysis of risk factors

Meta-analysis was performed for 14 risk factors, and 7 of these were statistical significance (*P* < 0.05). The risk factors analyzed in this review included trauma history (1.37 [95% CI 1.03–1.82], *P* = 0.030), BMI ≥ 24 kg/m^2^ (1.30 [95% CI 1.09–1.56], *P* = 0.004), gender (female) (1.04 [95% CI 1.00–1.09], *P* = 0.030), age (1.02 [95% CI 1.01–1.03], *P* = 0.007) and protective factors were exercise (0.75 [95% CI 0.62–0.91 *P* = 0.003), education background in high school (0.49 [95% CI 0.30–0.79], *P* = 0.003) and education background in university (0.22 [95% CI 0.06–0.86], *P* = 0.030) (Table 2). In the meta-analysis of cohort studies, the identified risk factors were BMI ≥ 24 kg/m^2^ (1.29 [95% CI 1.02–1.63], *P* = 0.030), age (1.04 [95% CI 1.02–1.06], *P* < 0.001) (Table 3). In case–control studies, protective factors included exercise (0.75 [95% CI 0.62–0.91]; *P* = 0.003), education background in high school (0.49 [95% CI 0.30–0.79]; *P* = 0.003) and education background in university (0.22 [95% CI 0.06–0.86], *P* = 0.030) (Table 4).

**Table 2:** Pooled ORs for association of commonly studied risk factors with knee OA (cohort study + case–control studies)

Risk factors	NO of studies	Pooled OR	Lower CI	Upper CI	*P* value	*I* squared (%)
Trauma history	6	1.37	1.03	1.82	0.030	0.0
BMI ≥ 24 kg/m2	11	1.30	1.09	1.56	0.004	0.0
Gender	17	1.04	1.00	1.09	0.030	20.0
Age	11	1.02	1.01	1.03	0.007	37.0
Exercise	5	0.75	0.62	0.91	0.003	25.0
High school	2	0.49	0.30	0.79	0.003	0.0
University	2	0.22	0.06	0.86	0.030	65.0
Living environment	3	2.05	0.66	6.34	0.210	0.0
Junior high school	2	1.21	0.62	2.36	0.570	0.0
Agricultural labour	2	1.17	0.54	2.56	0.690	0.0
Manual labour	7	1.10	0.96	1.26	0.160	0.0
Family history	4	1.04	0.98	1.10	0.220	0.0
Drinking	5	0.99	0.80	1.23	0.940	0.0
Smoking	7	0.93	0.81	1.07	0.330	0.0

**Table 3:** Pooled ORs for association of commonly studied risk factors with knee OA (cohort study)

Risk factors	NO of studies	Pooled OR	Lower CI	Upper CI	*P* value	*I* squared (%)
BMI ≥ 24 kg/m^2^	5	1.29	1.02	1.63	0.030	0.0
Age	4	1.04	1.02	1.06	< 0.001	0.0
Trauma history	4	1.34	0.99	1.82	0.060	0.0
Gender (female)	9	1.10	0.98	1.24	0.090	0.0
Manual labour	3	1.09	0.88	1.35	0.410	0.0
Drinking	3	0.98	0.79	1.21	0.860	0.0
Smoking	4	0.88	0.74	1.04	0.120	0.0

**Table 4:** Pooled ORs for association of commonly studied risk factors with knee OA (case–control studies)

Risk factors	NO of studies	Pooled OR	Lower CI	Upper CI	*P* value	*I* squared (%)
Exercise	5	0.75	0.62	0.91	0.003	25.0
High school	2	0.49	0.30	0.79	0.003	0.0
University	2	0.22	0.06	0.86	0.030	65.0
Living environment	3	2.05	0.66	6.34	0.210	0.0
Drinking	2	1.60	0.40	6.45	0.510	0.0
Trauma history	2	1.53	0.71	3.33	0.280	0.0
BMI ≥ 24 kg/m2	6	1.32	1.00	1.74	0.050	0.0
Junior high school	2	1.21	0.62	2.36	0.570	0.0
Agricultural labour	2	1.17	0.54	2.56	0.690	0.0
Manual labour	4	1.11	0.93	1.31	0.250	0.0
Smoking	3	1.09	0.84	1.41	0.530	0.0
Gender	8	1.05	0.99	1.12	0.090	55.0
Family history	4	1.04	0.98	1.10	0.220	0.0
Age	7	1.01	1.00	1.01	0.140	0.0

### Trauma history

**Fig. 2:**
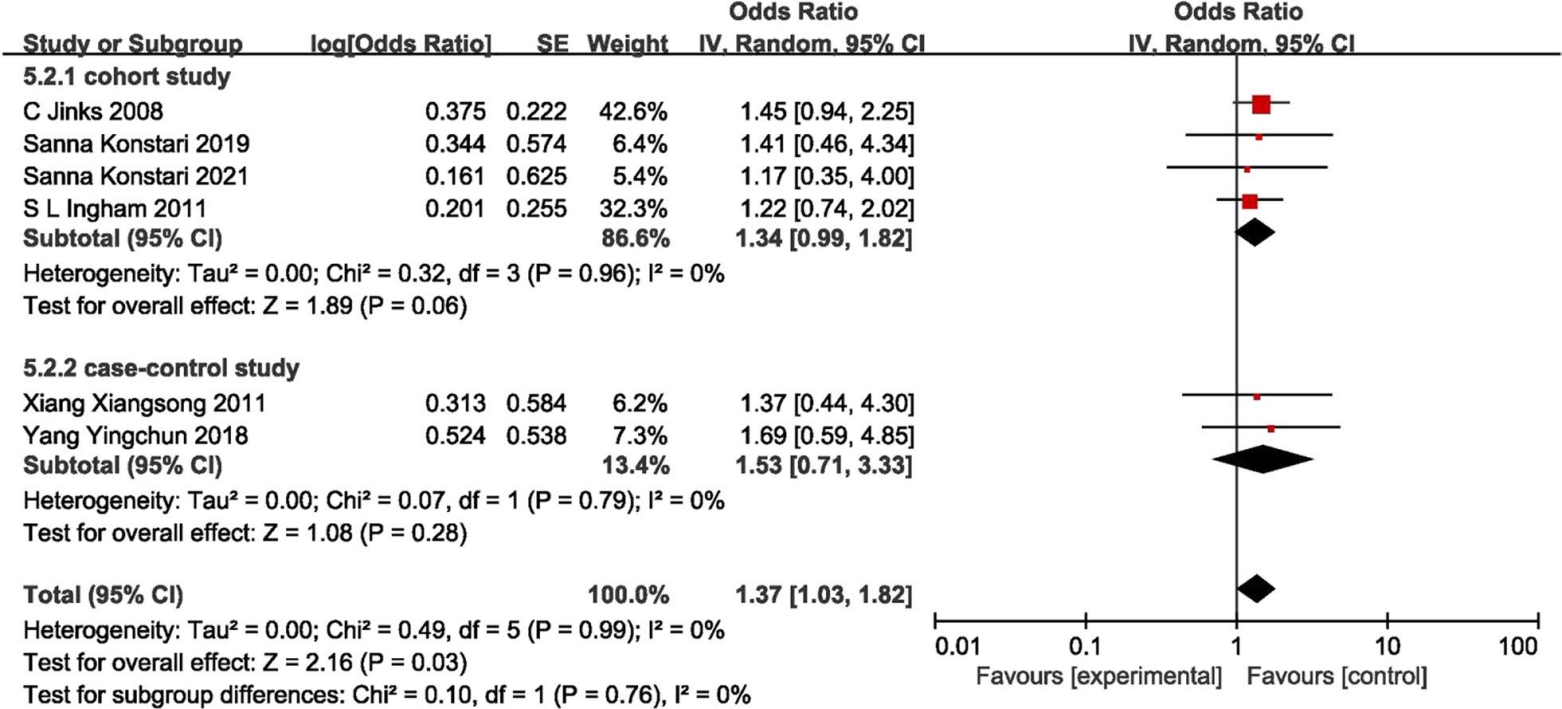
Forest plot of trauma history

Six studies investigated the trauma history of knee as a risk factor for onset of KOA in 29 included papers [12, 21, 22, 31, 33, 38]. The results showed that (*I*^2^ = 0%) a trauma history of knee is the risk factors of KOA (OR = 1.37, 95% CI 1.03–1.82, *P* = 0.030), which was the statistical significance maximum value (Fig. 2). But the subgroup analysis results suggest that the history of trauma did not demonstrate statistical significance in either the meta-analysis of cohort studies or the meta-analysis of case–control studies.

### ***BMI*** ≥ ***24 kg/m***^***2***^

**Fig. 3:**
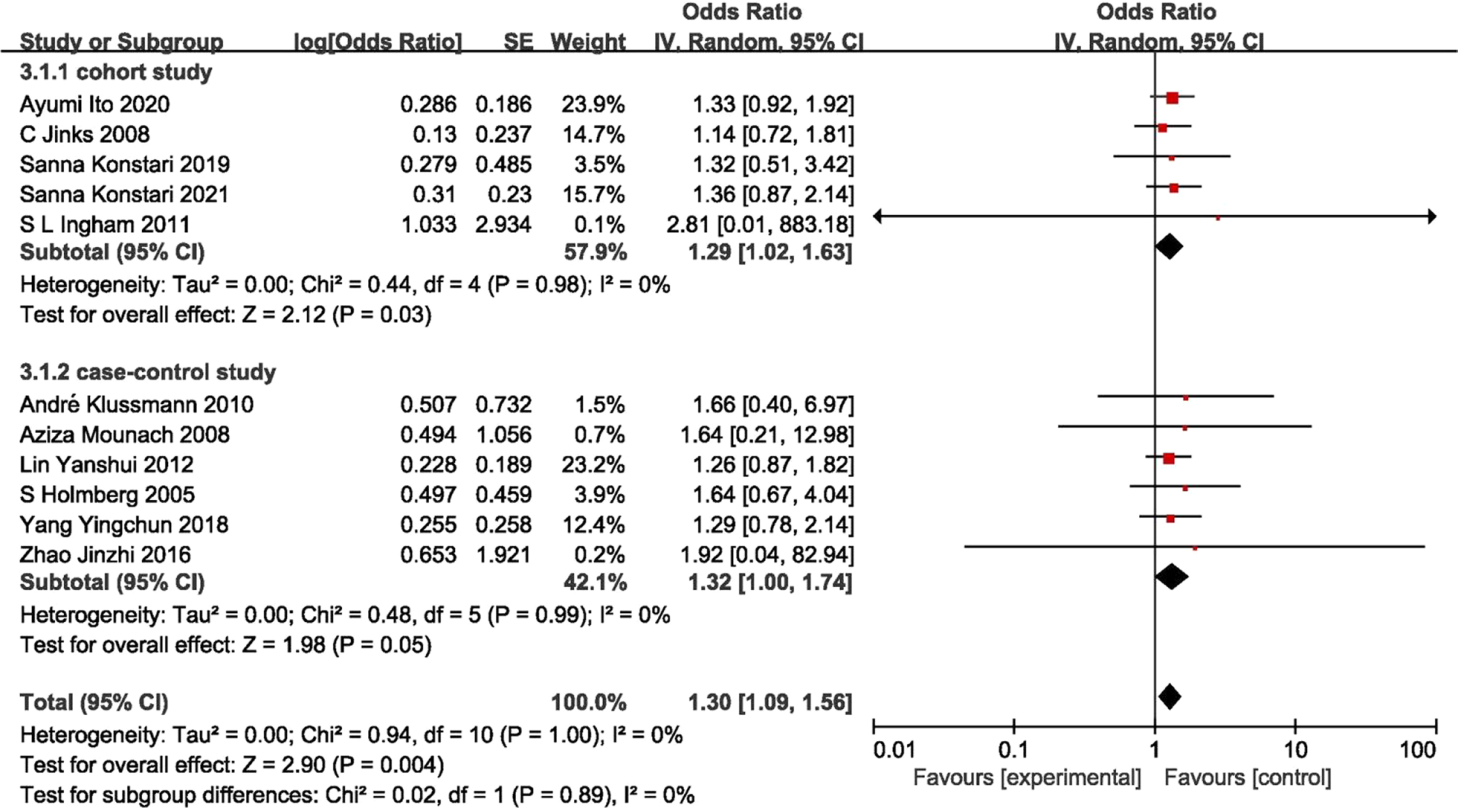
Forest plot of BMI

BMI ≥ 24 kg/m^2^ was demonstrated as the risk factors of onset of KOA in eleven included studies [12, 14, 21, 22, 24, 28, 33, 34, 37–39]. The pooled OR was 1.30 (95% CI 1.09–1.56), with *I*^2^ = 0%, *P* = 0.004. And the subgroup analysis revealed that while BMI was identified as a risk factor in the meta-analysis of cohort studies (1.29 [95% CI 1.02–1.63], *P* = 0.030), no significant difference was observed in the case–control studies (Fig. 3).

### Gender (female)

**Fig. 4:**
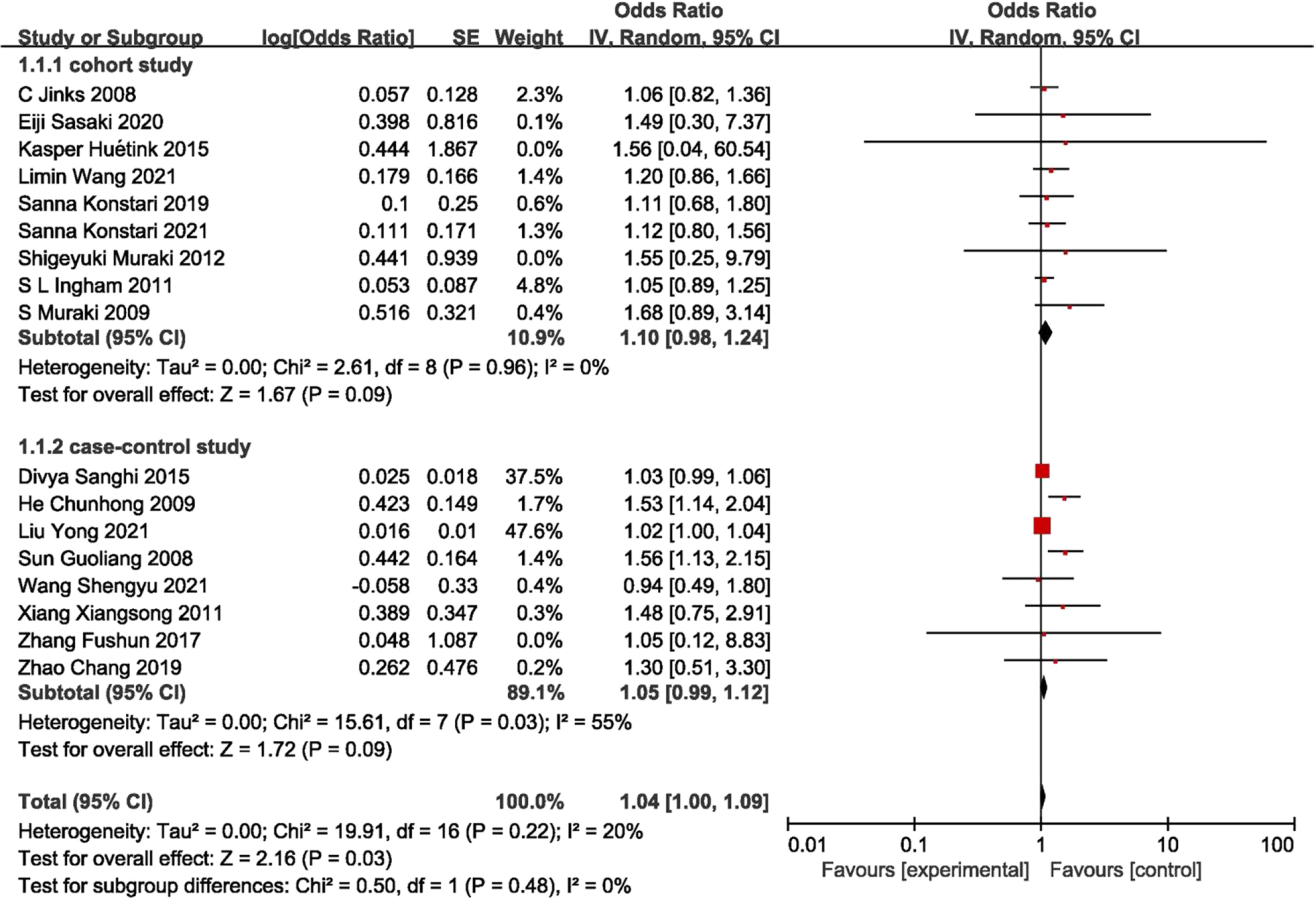
Forest plot of gender

In this study, the correlation between gender (female) and the onset of KOA was investigated, with 17 studies included [12, 13, 15–18, 21, 23, 25, 26, 29, 31, 33, 35, 36, 38, 40]. The pooled OR was 1.04 (95% CI 1.00–1.09), *I*^2^ = 20%, *P* = 0.030), showing that female was a risk factor for KOA, which was 1.04 times higher than that of male (Fig. 4). However, the results of the subgroup analysis indicate that neither the meta-analysis of cohort studies nor the meta-analysis of case–control studies could establish female gender as a risk factor for knee osteoarthritis.

### Age

**Fig. 5:**
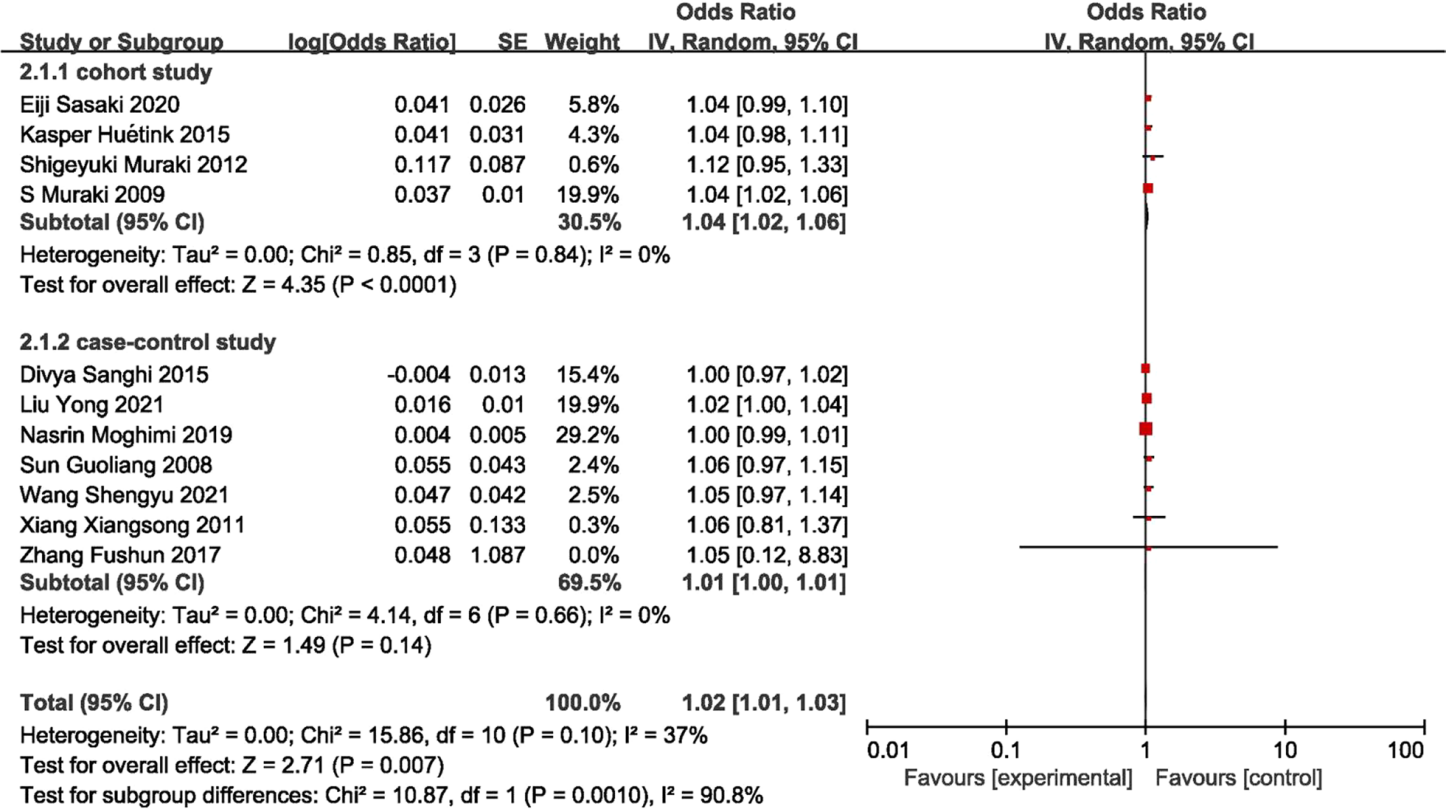
Forest plot of age

A total of 11 articles were included in the meta-analysis for the age [13, 16–19, 23, 25, 26, 29, 31, 36], with pooled OR 1.02 (95% CI 1.01–1.03, *I*^2^ = 37%, *P* = 0.007). In the subgroup analysis, the meta-analysis of cohort studies revealed that age is a contributing factor to the development of knee osteoarthritis (KOA) (1.04 [95% CI 1.02–1.06], *P* < 0.001), whereas the results of case–control studies indicated that age is not a significant pathogenic factor (Fig. 5).

### Educational background

**Fig. 6:**
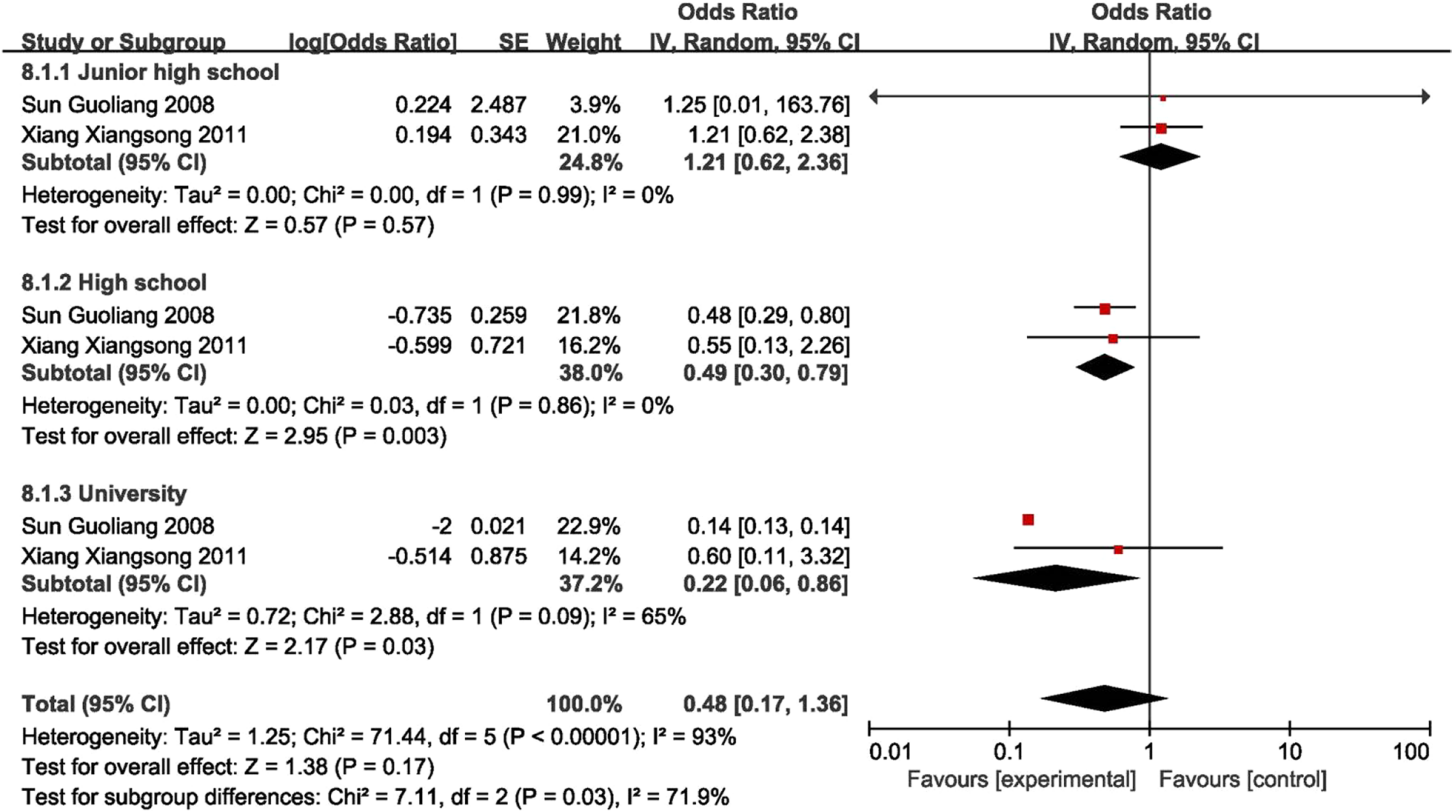
Forest plot of education level (case–control study)

Two case–control studies reported results on assessing the effect of being either junior high school or university [16, 31]. The findings indicate that completion of high school (0.49 [95% CI 0.30–0.79], *P* = 0.003) or university education (0.22 [95% CI 0.06–0.86], *P* = 0.030) serves as a protective factor against knee osteoarthritis (KOA), and this difference is statistically significant (Fig. 6).

### Eexercise

**Fig. 7:**
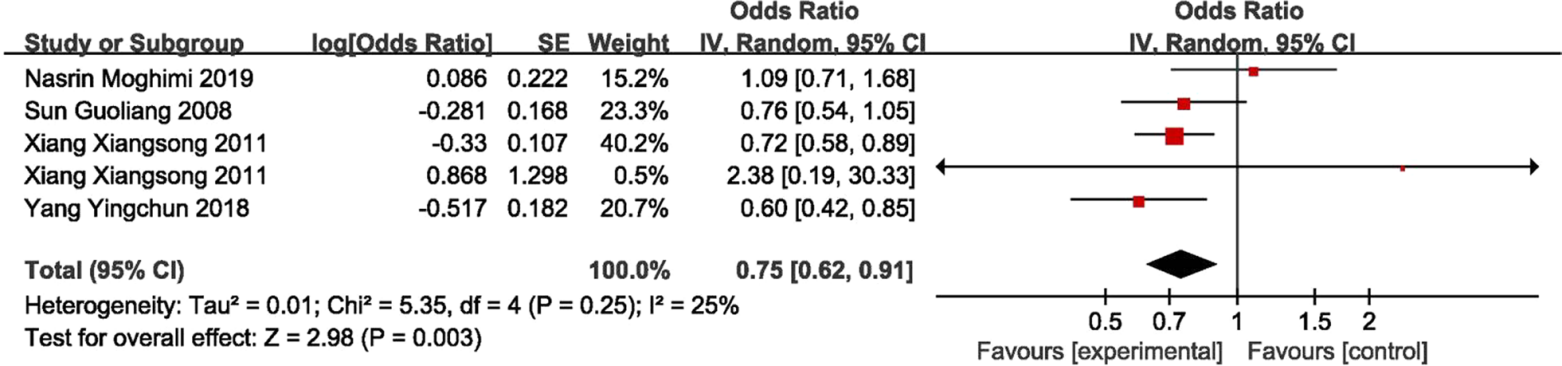
Forest plot of exercise (case–control study)

Four studies (one of them provided two types of exercise [31]) were included in the meta-analysis of exercise as a protective factors for onset of KOA [16, 19, 22, 31]. The pooled OR was 0.75 (95% CI 0.62–0.91). One of the articles [31] analyzed professional exercise and low-intensity exercise, which showed that low-intensity exercise was a protective factor for avoiding KOA, with an OR value of 0.72 (95% CI 0.58–0.89). (Fig. 7).

### Other factors

**Fig. 8:**
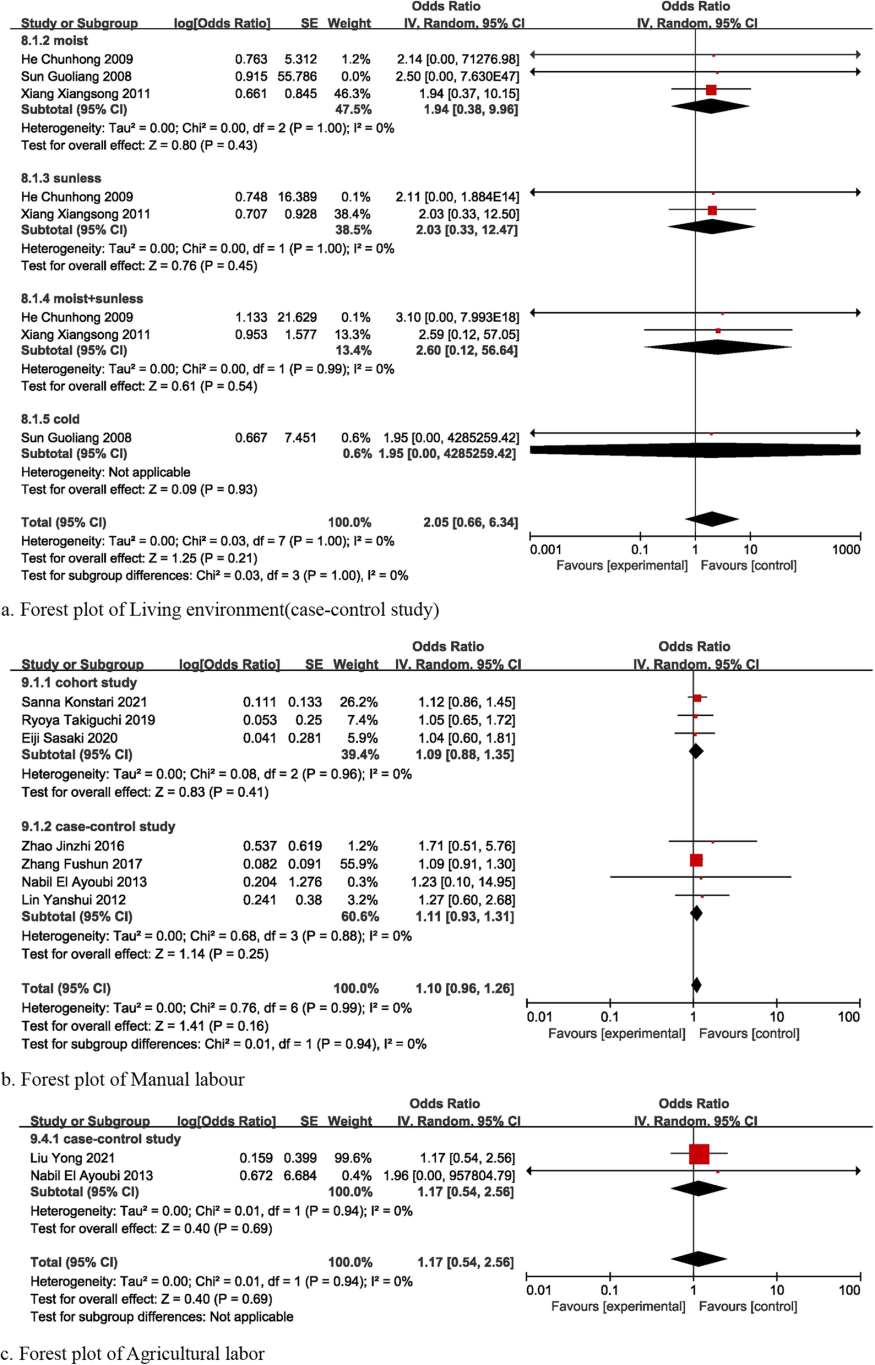

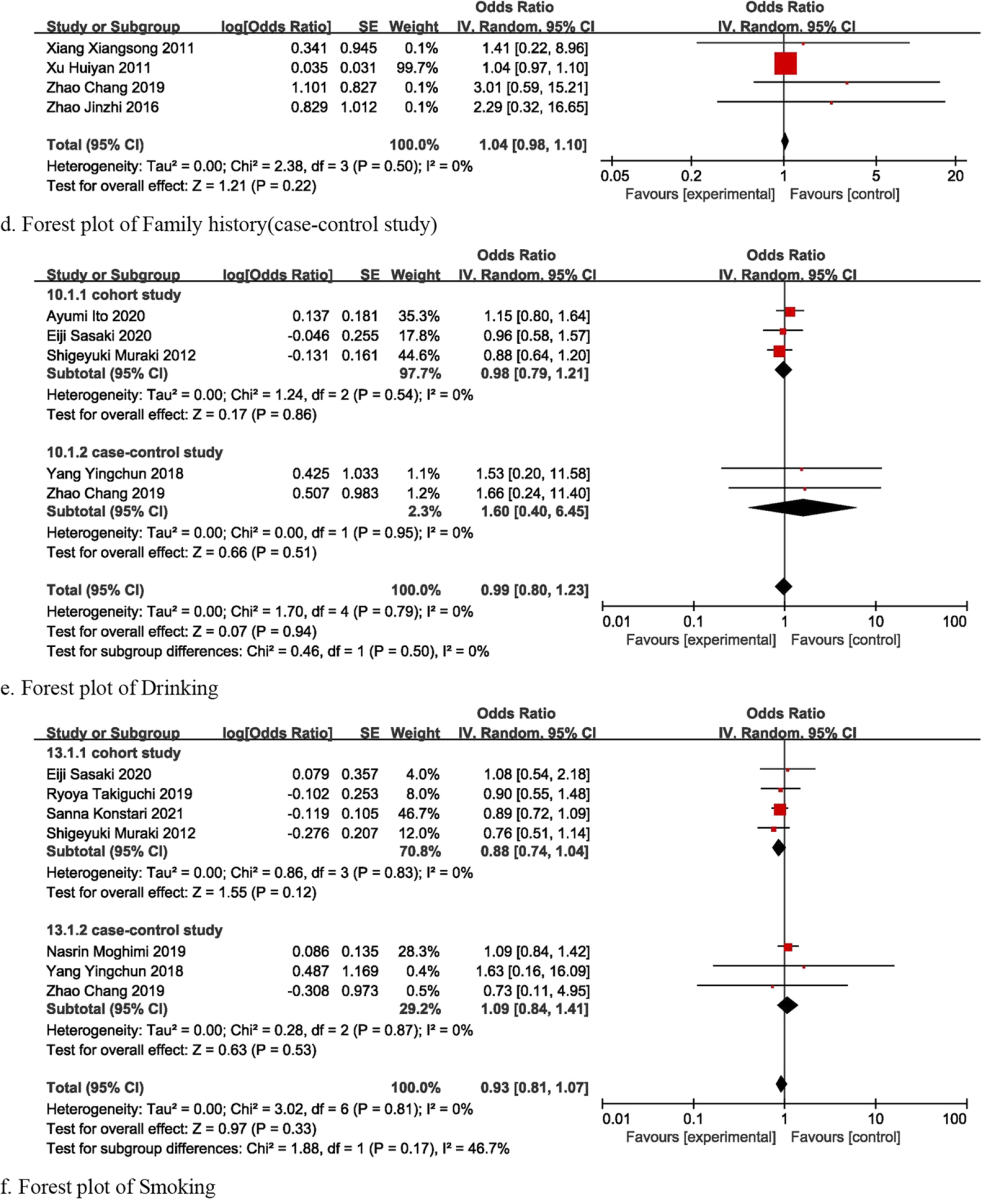
Forest plot of other factors

Meta-analysis was also conducted for other factors including living environment [16, 31, 35], work background [12, 13, 17, 20, 23, 24, 27, 28], family history [15, 24, 31, 32], smoking history [12, 13, 15, 19, 20, 22, 29], and drinking history [13–15, 22, 29]. Whether these factors were risk or protective factors for KOA cannot be proved in this review (Fig. 8a–f).

## Discussion

Knee osteoarthritis (KOA) is a chronic disease characterized by pain and joint dysfunction. Epidemiological studies have found that the incidence of KOA in Korea, the United Kingdom, Spain, and other regions for individuals over 40 years old has reached more than 15%. With the aging society, there has been a sequential rise in the incidence of KOA [41, 42]. However, current treatments are mainly symptomatic, including Chinese patent medicine, non-steroidal anti-inflammatory drugs, sodium hyaluronate injection, and hormone injection, which fail to reverse the disease [43]. This leads to tremendous pressure on medical resources due to long-term symptomatic treatment. Patients also experience poorer quality of life and higher financial burden due to the long-standing treatment, which ultimately leads to joint replacement and revision. Some researchers have demonstrated that risk factors for KOA include being overweight, female, having a history of joint injury, and age [3, 43]. However, some of these factors, such as age and gender, cannot be intervened at home. Meanwhile, new evidence has been updated with the progress of KOA-related research. Combining this new evidence to find the risk factors that patients can avoid at home and exploring protective factors can provide a basis for the development of KOA guidelines. This can help people delay or even avoid the onset of KOA and reduce the social burden caused by the disease. This review aims to explore the risk factors and protective factors of KOA onset through meta-analysis, which will benefit residents by helping them avoid the onset of KOA. According to the results, middle-aged and older individuals can delay or avert the occurrence of KOA at home by avoiding a history of trauma and high BMI, and by engaging in appropriate exercise and improving their educational background.

The history of knee trauma is widely recognized as a key factor in the development of knee osteoarthritis (KOA) by various researchers [38, 44]. Knee trauma can cause cartilage inflammation [43], alter the biomechanics of the knee joint [31], and ultimately lead to KOA, which is consistent with the findings of this review. Long-term overweight or obesity can result in muscle loss and fat accumulation, leading to the release of inflammatory factors that increase knee pressure or alter the biomechanics of the knee joint, ultimately inducing KOA [45]. Studies by Ayumi Ito et al. [14] have demonstrated that maintaining a lighter body weight over a period of 10 years can reduce the risk of KOA by 27.5%. Our results also indicate that body mass index (BMI) is a significant factor in the development of KOA. It is worth noting that both knee trauma and overweight can be prevented at home, as recommended by the 2019 American College of Rheumatology/Arthritis Foundation Guideline (ACR) [42]. Therefore, it is strongly recommended that patients control their weight at home to prevent the occurrence of KOA. Joint instability and cartilage defects, especially in women, can also contribute to the development of KOA with age, and the risk of KOA peaks around the age of 50 [13]. Our study shows that women are 1.04 times more likely to develop KOA than men, and middle-aged and elderly individuals over 40 are 1.02 times more likely to develop KOA than those under 40. Although age and gender cannot be altered, this information can still be used to identify specific groups that need to pay more attention to other risk factors. In addition to the four risk factors, this study has identified two protective factors: education level and exercise. The results indicate that individuals with a high school degree have a 0.49 times lower probability of developing KOA compared to those with a lower education level, while individuals with a college degree have a 0.22 times lower probability of developing KOA compared to those with a lower education level. Sun Guoliang et al. [16] also found that higher education is associated with a lower risk of KOA, possibly due to the reduced physical labor and lower risk of knee joint damage associated with higher education. With regards to exercise, many scholars believe that regular exercise can delay the onset of KOA. The 2019 Osteoarthritis Research Society International (OARSI) guidelines also recommend exercise as a preventative measure against KOA. However, it should be noted that while the 2019 American College of Rheumatology guidelines (ACR) strongly recommend exercise as a protective factor against KOA, they also emphasize the need for further research to determine the specific types of exercise that can protect the knee joint. Xiang Xiangsong’s study [31] suggests that an appropriate amount of exercise is protective against KOA, while excessive exercise can actually increase the risk of KOA. Recent systematic evaluations by Filippo Migliorini et al. [46–48] suggest that excessive exercise may lead to an early onset of knee osteoarthritis (KOA), while moderate exercise does not. However, there is no clear conclusion on whether appropriate exercise can protect against knee degeneration. Therefore, further research is needed to determine the optimal exercise prescription for preventing KOA. In addition to the aforementioned factors, this study also analyzed the impact of living environment, occupation, family history, comorbidities, smoking history, and alcohol consumption. However, the results did not indicate that these factors are pathogenic factors for KOA.

In this study, we aimed to minimize heterogeneity by analyzing each risk factor in two subgroups: cohort study and case–control study. The results showed that the trauma history and gender were not statistically significant in either subgroup. Age and BMI ≥ 24 kg/m^2^ had statistical differences only in the cohort study subgroup. However, due to limitations in the inclusion criteria of the articles, education and exercise were only analyzed in the case–control subgroup. The differences in results between these subgroups may be attributed to the lower quality of the articles and the insufficient number of articles in each subgroup. For instance, although BMI ≥ 24 kg/m^2^ did not show significance in the case–control subgroup, it was at the boundary of *P* = 0.05. Therefore, it is not appropriate to completely dismiss its potential to induce KOA. Further analysis is needed by incorporating new studies, which is also one of the limitations of this study.

Admittedly, it is important to acknowledge that our study has certain limitations. Firstly, while we included 29 papers with a total of 60,354 participants, the sample size varied across different factors such as living environment, smoking, drinking, and comorbidities. The sample size for some of these factors was small, and a larger sample size test is needed. Secondly, the heterogeneity of some research results exceeded 50% due to inconsistencies in the definition of the same factor among different researchers. Finally, although we categorized the included articles into two subgroups for case–control and cohort studies based on study type, the results were not consistent between the different subgroups. This may be due to the lower quality of articles and the insufficient number of articles included in each subgroup. As new studies emerge, this study needs to be updated further.

## Conclusion

Through a meta-analysis of cohort and case–control studies, this review demonstrated that a history of knee trauma, overweight or obesity, Gender (female), and over 40 years of age are risk factors for KOA, while a high school education or an university education background and more exercise are protective factors for KOA.

### Supplementary Information

**Additional file 1: Appendix 1.** Search Strategy for all databases.

**Additional file 2: Appendix 2.** The Assessment of quality for 17 case-control studies and 12 Cohort studies.

**Additional file 3: Appendix 3.** Grading of Recommendations Assessments, Development and valuation (GRADE) assessment.

## Data Availability

Data supporting the fndings of this study can be found in the article. The protocol of this systematic review and meta-analysis is available in the Prospective Register of Systematic Reviews (PROSPERO) under number CDR42022329710.

## Abbreviations

ACR: American College of Rheumatology
AHRQ: Agency for Healthcare Research and Quality
BMI: Body mass index
CI: Confidence interval
CNKI: China National Knowledge Infrastructure
GRADE: Grading of Recommendations Assessments, Development and Evaluation
K/L: Kellgren Lawrence
KOA: Knee osteoarthritis
Mesh: Medical Subject Headings
OA: Osteoarthritis
OARSI: Osteoarthritis Research Society International
OR: Odds ratios
SE: Standard error
VIP: Chinese Science and Technology Periodical Database
