# Supplementary material for: Evidence on risk factors for knee osteoarthritis in middle-older aged: a systematic review and meta analysis
Source: J Orthop Surg Res. 2023 Aug 29;18:634. doi: 10.1186/s13018-023-04089-6 (PMC10464102; doi:10.1186/s13018-023-04089-6)
Supplement: Supplementary file 1 — Additional file 1: Appendix 1. Search Strategy for all databases. [file 13018_2023_4089_MOESM1_ESM.pdf]

Table 1 Search strategy used in PubMed database.

Number	Search terms
1	knee osteoarthritis.ti, ab.
2	degenerative knee arthritis. ti, ab.
3	knee arthritis. ti, ab.
4	osteoarthritis of the knee. ti, ab.
5	degenerative knee osteoarthritis. ti, ab.
6	or 1-5
7	risk factor. ti, ab.
8	influence factor. ti, ab.
9	related factors. ti, ab.
10	pathogen. ti, ab.
11	Epidemiology. ti, ab.
12	or 7-10
13	cohort. ti, ab.
14	cohort study. ti, ab.
15	prospective. ti, ab.
16	prospective trial. ti, ab.
17	follow-up. ti, ab.
18	follow-up study. ti, ab.
19	or 12-17
20	6 and 11 and 18

Table 2 Search strategy used in Web of Science database.

Number	Search terms
1	knee osteoarthritis.ti, ab.
2	degenerative knee arthritis. ti, ab.
3	knee arthritis. ti, ab.
4	osteoarthritis of the knee. ti, ab.
5	degenerative knee osteoarthritis. ti, ab.
6	or 1-5
7	risk factor. ti, ab.
8	influence factor. ti, ab.
9	related factors. ti, ab.
10	pathogen. ti, ab.
11	Epidemiology. ti, ab.
12	or 7-10
13	cohort. ti, ab.
14	cohort study. ti, ab.
15	prospective. ti, ab.
16	prospective trial. ti, ab.
17	follow-up. ti, ab.
18	follow-up study. ti, ab.

19	or 12-17
20	6 and 11 and 18

Table 3 Search strategy used in OVID database.

Number	Search terms
1	knee osteoarthritis. ab.
2	degenerative knee arthritis. ab.
3	knee arthritis. ab.
4	osteoarthritis of the knee. ab.
5	degenerative knee osteoarthritis. ab.
6	or 1-5
7	risk factor. ab.
8	influence factor. ab.
9	related factors. ab.
10	pathogen. ab.
11	Epidemiology. ab.
12	or 7-10
13	cohort. ab.
14	cohort study. ab.
15	prospective. ab.
16	prospective trial. ab.
17	follow-up. ab.
18	follow-up study. ab.
19	or 12-17
20	6 and 11 and 18

Table 4 Search strategy used in CNKI database.

Number	Search terms
1	xiguguanjieyan. tka.
2	xiguanjieguguanjieyan. tka.
3	xiguanjieguxingguanjieyan. tka.
4	Xiguxingguanjieyan. tka.
5	or 1-4
6	weixianyinsu. tka.
7	yingxiangyinsu. tka.
8	xiangguanyinsu. tka.
9	or 6-8
10	5 and 9

Table 5 Search strategy used in Wanfang database.

Number	Search terms
1	xiguguanjieyan. su.
2	xiguanjieguguanjieyan. su.
3	xiguanjieguxingguanjieyan. su.
4	Xiguxingguanjieyan. su.
5	or 1-4
6	weixianyinsu. su.
7	yingxiangyinsu. su.
8	xiangguanyinsu. su.
9	or 6-8
10	5 and 9

Table 6 Search strategy used in Chongqing VIP database.

Number	Search terms
1	xiguguanjieyan. ti.
2	xiguanjieguguanjieyan. ti.
3	xiguanjieguxingguanjieyan. ti.
4	Xiguxingguanjieyan. ti.
5	or 1-4
6	weixianyinsu. ti.
7	yingxiangyinsu. ti.
8	xiangguanyinsu. ti.
9	or 6-8
10	5 and 9

Table 7 Search strategy used in SinoMed database.

Number	Search terms
1	xiguguanjieyan. ti,ab,kw,su.
2	xiguanjieguguanjieyan. ti,ab,kw,su.
3	xiguanjieguxingguanjieyan. ti,ab,kw,su.
4	Xiguxingguanjieyan. ti,ab,kw,su.
5	or 1-4
6	weixianyinsu. ti,ab,kw,su.
7	yingxiangyinsu. ti,ab,kw,su.
8	xiangguanyinsu. ti,ab,kw,su.
9	or 6-8
10	5 and 9
