# Supplementary material for: Evidence on risk factors for knee osteoarthritis in middle-older aged: a systematic review and meta analysis
Source: J Orthop Surg Res. 2023 Aug 29;18:634. doi: 10.1186/s13018-023-04089-6 (PMC10464102; doi:10.1186/s13018-023-04089-6)
Supplement: Supplementary file 2 — Additional file 2: Appendix 2. The Assessment of quality for 17 case-control studies and 12 Cohort studies. [file 13018_2023_4089_MOESM2_ESM.pdf]

Appendix A The Assessment of quality for 17 case-control studies

Study	Selection				Comparability	Exposure			Scores
	Is the case definition adequate?	Representativeness of the cases	Selection of Controls	Definition of Controls	Comparability of cases and controls on the basis of the design or analysis	Ascertainment of exposure	Same method of ascertainment for cases and controls	Non-Respon se rate	
HuiYan Xu 2011[32]	★	★	★	★	★	★	★	☆	7
XiangXiang Song 2011[31]	★	★	☆	★	★	☆	★	☆	5
YinChun Yang 2018[22]	★	★	☆	★	★	★	★	★	7
GuoLiang Sun 2008[16]	★	★	☆	★	★	★	★	★	7
ShengYu Wang 2021[18]	★	★	★	★	★★	★	★	☆	8
ChunHong He 2009[35]	★	★	☆	★	★	★	★	☆	6
Chang Zhao 2019[15]	★	★	☆	★	★	☆	★	☆	5
YanShui Lin 2012[28]	☆	★	☆	★	☆☆	☆	★	☆	3

JinZhi Zhao 2016[24]	☆	★	☆	★	☆☆	★	★	☆	4
FuShun Zhang 2017[23]	☆	★	☆	★	☆☆	☆	★	☆	3
Yong Liu 2021[17]	★	★	☆	★	☆☆	★	★	☆	4
Nasrin Moghimi 2019[19]	★	★	☆	★	★	★	★	☆	5
Aziza Mounach 2008[37]	★	★	☆	★	★★	★	★	☆	7
Nabil El Ayoubi 2013[27]	★	★	☆	★	★	★	★	☆	6
Holmberg S 2005[39]	★	★	★	★	★★	☆	★	☆	7
Klussmann A 2010[34]	★	★	★	★	★	☆	★	☆	6
Divya Sanghi 2015[26]	★	★	★	★	★	★	★	☆	7

Appendix B The Assessment of quality for 12 Cohort studies

Study	Selection				Comparability	Exposure			Scores
	Representativeness of the exposed cohort	Selection of the non exposed cohort	Ascertainment of exposure	Demonstration that outcome of interest was not present at start of study	Comparability of cohorts on the basis of the design or analysis	Assessment of outcome	Was follow-up long enough for outcomes to occur	Adequacy of follow up of cohorts	
C Jinks 2008[38]	★	★	★	★	★	★	★	★	8
Ryoya Takiguchi 2019[20]	★	★	☆	★	★★	★	★	☆	7
Ingham SL 2011[33]	★	★	☆	★	★	★	★	☆	6
Muraki S 2012[29]	★	★	☆	★	★	★	★	☆	6
Huétink K 2015[25]	★	★	☆	★	★	★	★	★	7
Eiji Sasaki 2020[13]	★	★	☆	★	★	★	☆	☆	5
Ito A 2020[14]	★	★	★	★	★★	★	★	☆	8
Yoshimura N 2012[30]	★	★	★	★	★	★	★	★	8
Konstari S	★	★	★	★	★★	★	★	★	9

2021[12]									
Wang L 2021[40]	★	★	★	★	★	★	★	☆	7
Konstari S 2019[21]	★	★	☆	★	★	★	★	☆	6
Muraki S 2009[36]	★	★	★	★	★	★	★	☆	7
