# Supplementary material for: Evidence on risk factors for knee osteoarthritis in middle-older aged: a systematic review and meta analysis
Source: J Orthop Surg Res. 2023 Aug 29;18:634. doi: 10.1186/s13018-023-04089-6 (PMC10464102; doi:10.1186/s13018-023-04089-6)
Supplement: Supplementary file 3 — Additional file 3: Appendix 3. Grading of Recommendations Assessments, Development and valuation (GRADE) assessment. [file 13018_2023_4089_MOESM3_ESM.pdf]

Author(s):
Question: Risk factors in KOA
Setting:
Bibliography:

Certainty assessment							№ of patients		Effect		Certainty	Importance
№ of studies	Study design	Risk of bias	Inconsistency	Indirectness	Imprecision	Other considerations	Risk factors		Relative (95% CI)	Absolute (95% CI)		
**gender - cohort study**												
9	observational studies	serious[a]	not serious	not serious	not serious	publication bias strongly suspected[b]	6654/30087 (22.1%)	0/0	**OR 1.10** (0.98 to 1.24)	**1 fewer per 1,000** (from 1 fewer to 1 fewer)	⊕◯◯◯ Very low	CRITICAL
**gender - case-control study**												
8	observational studies	serious[a]	serious[c]	not serious	not serious	publication bias strongly suspected[b]	2674 cases 2829 controls<br>-	 0.0%	**OR 1.05** (0.99 to 1.12)	-<br>**0 fewer per 1,000** (from 0 fewer to 0 fewer)	⊕◯◯◯ Very low	CRITICAL
**age - cohort study**												
4	observational studies	serious[a]	not serious	not serious	not serious	none	4014/6413 (62.6%)	-/0	**OR 1.04** (1.02 to 1.06)	**1 fewer per --** (from 1 fewer to 1 fewer)	⊕◯◯◯ Very low	CRITICAL
**age - case-control study**												
7	observational studies	serious[a]	not serious	not serious	not serious	none	3098 cases 3243 controls<br>-	 0.0%	**OR 1.01** (1.00 to 1.01)	-<br>**0 fewer per --** (from 0 fewer to 0 fewer)	⊕◯◯◯ Very low	CRITICAL
**BMI - cohort study**												
5	observational studies	serious[a]	not serious	not serious	not serious	publication bias strongly suspected[b]	4085/7797 (52.4%)	-/0	**OR 1.06** (1.04 to 1.09)	**1 fewer per 1,000** (from 1 fewer to 1 fewer)	⊕◯◯◯ Very low	CRITICAL
**BMI - case-control study**												
6	observational studies	serious[a]	serious[c]	not serious	not serious	publication bias strongly suspected[b]	2765 cases 3183 controls<br>-	 0.0%	**OR 1.04** (1.00 to 1.08)	-<br>**0 fewer per --** (from 0 fewer to 0 fewer)	⊕◯◯◯ Very low	CRITICAL
**BMI≥24kg/m2 - cohort study**												
6	observational studies	serious[a]	not serious	not serious	not serious	publication bias strongly suspected[b]	3106/22915 (13.6%)	-/0	**OR 1.29** (1.02 to 1.63)	**1 fewer per --** (from 2 fewer to 1 fewer)	⊕◯◯◯ Very low	CRITICAL
**BMI≥24kg/m2 - case-control study**												
5	observational studies	serious[a]	not serious	not serious	not serious	publication bias strongly suspected[b]	1974 cases 1734 controls<br>-	 0.0%	**OR 1.32** (1.00 to 1.74)	-<br>**0 fewer per --** (from 0 fewer to 0 fewer)	⊕◯◯◯ Very low	CRITICAL
**Trauma history - case-control study**												
2	observational studies	serious[a]	not serious	not serious	not serious	publication bias strongly suspected[b]	1517 cases 1648 controls<br>-	 0.0%	**OR 1.53** (0.71 to 3.33)	-<br>**0 fewer per --** (from 0 fewer to 0 fewer)	⊕◯◯◯ Very low	IMPORTANT
**Trauma history - cohort study**												
4	observational studies	serious[a]	not serious	not serious	not serious	publication bias strongly suspected[b]	1825/15481 (11.8%)	-/0	**OR 1.34** (0.99 to 1.82)	**1 fewer per --** (from 2 fewer to 1 fewer)	⊕◯◯◯ Very low	IMPORTANT

**case-control study - moist**												
3	observational studies	serious[a]	not serious	not serious	not serious	publication bias strongly suspected[b]	1622 cases 1768 controls		**OR 1.94** (0.38 to 9.96)	-	⨁◯◯◯ Very low	NOT IMPORTANT
							-	0.0%		**0 fewer per --** (from 0 fewer to 0 fewer)		
**case-control study - sunless**												
2	observational studies	serious[a]	not serious	not serious	not serious	publication bias strongly suspected[b]	1506 cases 1621 controls		**OR 2.03** (0.33 to 12.47)	-	⨁◯◯◯ Very low	NOT IMPORTANT
							-	0.0%		**0 fewer per --** (from 0 fewer to 0 fewer)		
**case-control study - moist+sunless**												
2	observational studies	serious[a]	not serious	not serious	not serious	publication bias strongly suspected[b]	1506 cases 1621 controls		**OR 2.60** (0.12 to 56.64)	-	⨁◯◯◯ Very low	NOT IMPORTANT
							-	0.0%		**0 fewer per --** (from 0 fewer to 0 fewer)		
**case-control study - cold**												
1	observational studies	serious[a]	not serious	not serious	not serious	publication bias strongly suspected[b]	116 cases 147 controls		**OR 1.95** (0.00 to 4285259.42)	-	⨁◯◯◯ Very low	NOT IMPORTANT
							-	0.0%		**0 fewer per --** (from 0 fewer to --)		
**case-control study - Junior high school**												
2	observational studies	serious[a]	not serious	not serious	not serious	publication bias strongly suspected[b]	1496 cases 1647 controls		**OR 1.21** (0.62 to 2.36)	-	⨁◯◯◯ Very low	NOT IMPORTANT
							-	0.0%		**0 fewer per --** (from 0 fewer to 0 fewer)		
**case-control study - High school**												
2	observational studies	serious[a]	not serious	not serious	not serious	publication bias strongly suspected[b]	1496 cases 1647 controls		**OR 0.49** (0.30 to 0.79)	-	⨁◯◯◯ Very low	NOT IMPORTANT
							-	0.0%		**0 fewer per --** (from 0 fewer to 0 fewer)		
**case-control study - University**												
2	observational studies	serious[a]	serious[d]	not serious	not serious	publication bias strongly suspected[b]	1496 cases 1647 controls		**OR 0.22** (0.06 to 0.86)	-	⨁◯◯◯ Very low	NOT IMPORTANT
							-	0.0%		**0 fewer per --** (from 0 fewer to 0 fewer)		
**case-control study - OA family history**												
4	observational studies	serious[a]	not serious	not serious	not serious	publication bias strongly suspected[b]	1800 cases 2223 controls		**OR 1.04** (0.98 to 1.10)	-	⨁◯◯◯ Very low	IMPORTANT
							-	0.0%		**0 fewer per 1,000** (from 0 fewer to 0 fewer)		
**case-control study - manual labour**												
4	observational studies	serious[a]	not serious	not serious	not serious	publication bias strongly suspected[b]	720 cases 783 controls		**OR 1.11** (0.93 to 1.31)	-	⨁◯◯◯ Very low	IMPORTANT
							-	0.0%		**0 fewer per 1,000** (from 0 fewer to 0 fewer)		
**case-control study - agricultural labor**												
2	observational studies	serious[a]	not serious	not serious	not serious	publication bias strongly suspected[b]	269 cases 323 controls		**OR 1.17** (0.54 to 2.56)	-	⨁◯◯◯ Very low	IMPORTANT
							-	0.0%		**0 fewer per 1,000** (from 0 fewer to 0 fewer)		

**cohort study - manual labour**												
3	observational studies	serious[a]	not serious	not serious	not serious	none	1272/18346 (6.9%)	-/0	**OR 1.09** (0.88 to 1.35)	**1 fewer per 1,000** (from 1 fewer to 1 fewer)	⨁◯◯◯ Very low	IMPORTANT
**cohort study - drinking**												
3	observational studies	serious[a]	not serious	not serious	not serious	publication bias strongly suspected[b]	3563/10710 (33.3%)	-/0	**OR 0.98** (0.79 to 1.21)	**1 fewer per 1,000** (from 1 fewer to 1 fewer)	⨁◯◯◯ Very low	NOT IMPORTANT
**case-control study - drinking**												
2	observational studies	serious[a]	not serious	not serious	not serious	none	287 cases 313 controls<br>-	 0.0%	**OR 1.60** (0.40 to 6.45)	-<br>**0 fewer per 1,000** (from 0 fewer to 0 fewer)	⨁◯◯◯ Very low	NOT IMPORTANT
**cohort study - smoking**												
4	observational studies	serious[a]	not serious	not serious	not serious	none	3170/20608 (15.4%)	-/0	**OR 0.88** (0.74 to 1.04)	**1 fewer per 1,000** (from 1 fewer to 1 fewer)	⨁◯◯◯ Very low	NOT IMPORTANT
**case-control study - smoking**												
3	observational studies	serious[a]	not serious	not serious	not serious	publication bias strongly suspected[b]	987 cases 1013 controls<br>-	 0.0%	**OR 1.09** (0.84 to 1.41)	-<br>**0 fewer per 1,000** (from 0 fewer to 0 fewer)	⨁◯◯◯ Very low	NOT IMPORTANT
**case-control study - exercise**												
4	observational studies	serious[a]	not serious	not serious	not serious	publication bias strongly suspected[b]	3713 cases 3995 controls<br>-	 0.0%	**OR 0.75** (0.62 to 0.91)	-<br>**0 fewer per 1,000** (from 0 fewer to 0 fewer)	⨁◯◯◯ Very low	IMPORTANT

CI: confidence interval; **OR:** odds ratio

Explanations

a. Most information is from studies at low or unclear risk of bias. Potential limitations are likely to lower confidence in the estimate of effect.
b. Funnel plot asymmetry
c. I^2=55%
d. I^2=65%
